# Joint Modeling and Operational Optimization of a Reverse Osmosis–Mechanical Vapor Recompression System for Coal-Fired Power Plant Wastewater

**DOI:** 10.3390/membranes14030065

**Published:** 2024-03-04

**Authors:** Fengling Xie, Yan Zhao, Aipeng Jiang, Rui Zhao, Chuang Li, Jian Wang

**Affiliations:** 1 School of Automation, Hangzhou Dianzi University, Hangzhou 310018, China; 211060052@hdu.edu.cn (F.X.); 222060184@hdu.edu.cn (R.Z.); lichuang_1006@163.com (C.L.); wj@hdu.edu.cn (J.W.); 2 CHN Energy Water and Environmental Protection Co., Ltd., Beijing 100039, China; 12030975@chnenergy.com.cn

**Keywords:** coal-fired plant wastewater, reverse osmosis (RO), mechanical vapor recompression (MVR), optimization, mechanism model, operational cost

## Abstract

The operation of coal-fired power plants generates a large amount of wastewater. With the issuance of increasingly strict drainage standards, the cost of wastewater treatment is increasing, and the need to reduce the cost of wastewater treatment is becoming increasingly urgent. Thus, based on the principles of reverse osmosis (RO) and mechanical vapor recompression (MVR) in wastewater treatment, the operational optimization of an RO-MVR joint system was studied in this work with the consideration of reducing the operating costs of wastewater treatment under given operational conditions. Firstly, based on the basic principles of RO and MVR, corresponding mechanism models were established and their accuracy was verified. Then, an economic model of the RO-MVR joint system was established, with the goal of minimizing the water production unit price and daily operating costs of the joint system for optimization analysis. Finally, we analyzed the cost and water production performance of the RO-MVR joint system before and after optimization under different operating conditions. The results show that this optimization based on the RO-MVR joint system will reduce the unit price of water production to 3.16 CNY/m^3^, with the daily operating costs being decreased by 22% compared to before optimization. This result helps to reduce the cost of zero-discharge wastewater treatment in coal-fired power plants.

## 1. Introduction

Coal power plants generate a large amount of high-salinity wastewater, and direct discharge of this wastewater can pollute water quality, harm aquatic organisms, and disrupt the safety of local drinking water [1,2,3]. In the past, the traditional method of centralized collection and treatment of wastewater has been proven to further increase the complexity, volatility, and toxicity of wastewater components, and cause secondary pollution problems [4]. With the continuous development of industrial wastewater treatment technology, concentration technology and crystallization technology are widely used in the treatment of high-salinity wastewater [3].

Reverse osmosis (RO) technology is a typical concentration technology, which is simple and reliable in operation, has a high desalination rate, and exhibits a wide range of water quality [5,6]. Nearly 80% of seawater desalination plants in the world use RO technology [7]. Jafarinejad [8] found in their study that RO is a feasible technique for treating oily wastewater, and that the desalination rate of oily wastewater using reverse osmosis membranes can reach 99% or even higher. Belkacem et al. [9] found that RO technology is very suitable for the treatment of pharmaceutical wastewater, with a retention of over 99% for the totality of solutes, which meets the standards of the pharmaceutical industry. Lee et al. [10] used polyamide RO membranes to reuse steel wastewater, demonstrating that RO technology can successfully remove dissolved monovalent and divalent ions from steel wastewater. As mentioned above, RO technology has been applied to fields such as petrochemical wastewater, pharmaceutical wastewater, and steelmaking wastewater [11,12,13].

Mechanical vapor recompression (MVR) is an advanced crystallization technology with high thermal efficiency, low operating costs, and low energy consumption [14,15]. It is an effective method for treating high-salinity wastewater. In the MVR seawater desalination plant built by Gee et al. [16], after applying desalination technology, the magnesium content is steadily reduced, which not only has high efficiency but also is very reliable. Liang et al. [17] studied a dual-effect MVR evaporation system. Compared with a single-effect MVR system, the dual-effect MVR system has lower power consumption and can be used to treat wastewater with a high concentration of inorganic salt while saving energy. Kang et al. [18] designed an MVR evaporation system capable of treating a sodium chloride–potassium chloride water system, providing a new approach for low-energy separation of mixed-salt wastewater.

The coupling of RO technology and MVR technology can effectively remove ions and soluble solids from wastewater through physical filtration and concentration, clean wastewater, and avoid the secondary pollution problem caused by traditional chemical treatment of wastewater [19]. At the same time, the high-concentration saline water generated by the RO process is evaporated and crystallized to obtain mineral salts such as NaCl and Na_2_SO_4_, achieving high resource recovery and utilization. Ray et al. [20] investigated the total production costs of several membrane-based hybrid processes for the processing of corn steel water. They found that compared to the individual RO process, the RO-MVR method achieved optimal total production costs with 60% water removal through RO. Zhang et al. [21] applied MVR technology to RO systems when treating domestic wastewater, and found that compared with the original technology, the water quality of the treated system was greatly improved. The coupling of RO and MVR technology has become an attractive research direction for the zero-discharge treatment of high-salinity wastewater.

In the coupled system of RO and MVR, RO has a high operating pressure and high energy consumption, and fluctuations in feed conditions can greatly affect its performance and cost. The performance and cost of the MVR process are also affected by the RO process, which is very complex. Therefore, establishing a mechanism model for the coupled system of RO and MVR, and conducting research on this basis, is of great significance for reducing system operating costs and improving system performance, which can better meet production needs and achieve optimized control of the process. In terms of RO technology, Hermans [22] developed a model for hollow-fiber membranes and discussed the influence of flowrate and pressure during the RO process. Building upon Hermans’ work [22], Al-Bastaki et al. [23] improved the mechanistic model of hollow-fiber RO membranes based on solute diffusion theory, considering the impacts of pressure drop and concentration polarization phenomena, and the model’s accuracy was validated. Kaghazchi et al. [24] established a mathematical mechanism model for the RO process based on the research background of two desalination plants using Dow RO membrane SW30HR-380 in the Gulf region. A comparison with on-site data showed that this model has high accuracy and can be used to analyze the impact of operating parameters on water production performance. He et al. [25] established a mathematical model for the RO process to address the issues of opacity and high energy consumption in wastewater recovery in coal-fired power plants. They conducted research on the simulation and operational optimization of wastewater treatment in coal-fired power plants, aiming to facilitate the digitization of wastewater treatment systems. In terms of MVR technology, Aybar [26] introduced in detail the mechanism model of the evaporator in a MVR system based on the theory of overall energy conservation, mass conservation, and heat exchange, and studied the operating characteristics of a low-temperature mechanical steam compression system. In 2013, Shi et al. [27] proposed a technical scheme combining MVR evaporation with horizontal falling film evaporation. A mathematical model of the system was established based on energy and mass conservation theory, and the effects of evaporation temperature, feed solution temperature, and heat transfer temperature on the system’s performance were analyzed. Yue et al. [28], based on the work of Shi et al. [27], established a thermodynamic model for mechanical steam compression seawater desalination. They used equation solvers to analyze the effects of evaporation temperature and feed seawater concentration on system performance. In 2016, Onishi et al. [29] developed an optimization model for the rigorous design of SEE/MEE-MVR systems with thermal integration, aimed at achieving ZLD operations. They considered the estimation of the principal thermal and geometrical characteristics of the evaporation system, and studied the most cost-effective SEE/MEE-MVR process design to achieve ZLD conditions. In 2022, Shen et al. [30] proposed a novel double-effect staged MVR system. They used Aspen Plus simulation software (version 11) to build a model of the new double-effect staged MVR system and conducted simulation analysis on its energy consumption, operating costs, and compressor operating parameters. In terms of the RO-MVR joint system, Xiao et al. [31] proposed a UF-NF-RO-MVR hybrid system for zero-discharge treatment of pigment wastewater and studied the investment cost of this process. Their results showed that resource recovery and equipment investment cost reduction can be achieved by updating existing water treatment facilities. Wenzlick et al. [32] also discovered in their research on the treatment of water from oil and gas productions that the total salt production cost using NF-RO-MVR technology is 33% lower than when using MVR alone. Wang et al. [33] proposed the ED-RO-MVR integrated process and conducted energy analysis to achieve full utilization of materials. Their results showed that this process can achieve high-value conversion of wastewater. The above research confirms the superiority of the RO-MVR joint system and makes some improvements to it. At the same time, it provides strong support for the mechanism modeling of the RO-MVR joint system. However, there have been no reports on an economic cost analysis of the RO-MVR joint system model.

The above research provides strong support for the mechanism modeling of the RO-MVR joint system and confirms the advantages and prospects of the RO-MVR joint system. However, most studies mainly focus on seawater desalination processes or only on individual processes, and there is little modeling research on the entire process of zero-discharge wastewater treatment systems. In order to better guide operation optimization of the RO-MVR joint system, there is still some work to be carried out, especially for factories that need to improve their RO or MVR processes to achieve higher profits. Therefore, in order to achieve a reduction in operating costs in the treatment of coal-fired power plant wastewater, this article establishes the mechanism models of the RO process and MVR process, and also establishes an economic model of the RO-MVR joint system. It studies an optimization of operation for the RO-MVR joint system and analyzes the economic cost of coal power plants before and after optimization under different operating conditions. This mathematical model can also be applied to other similar applications, and our work helps to reduce the economic cost of coal chemical wastewater treatment.

## 2. Materials and Methods

### 2.1. Mechanism Model of the RO Process

**Figure 1:**
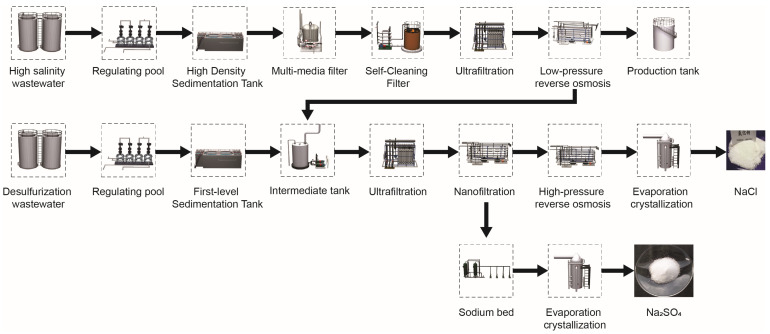
Process flow for the treatment of coal-fired wastewater.

This study focuses on wastewater from a coal-fired process in Inner Mongolia, as shown in Figure 1. The objective of this process is to achieve “zero discharge” of the wastewater. The main modules of the “zero discharge” system are RO and evaporation crystallization technologies. This paper primarily investigates the high-pressure RO module within the RO process and the evaporation crystallization module. The high-pressure RO process consists of one stage and three stages. The membrane element of the process is the high-pressure RO membrane element INDUSTRIAL RO7 8040F35 from Suez Company. The proportion of pressure vessels in the first, second, and third stages is 4:2:2, and each pressure vessel has six membrane elements. The evaporation crystallization module uses an MVR process to further concentrate and crystallize the RO-concentrated water. The coal power plant studied in this study has a total of ten devices. This article takes a single RO-MVR joint system as an example for research and calculation.

**Figure 2:**
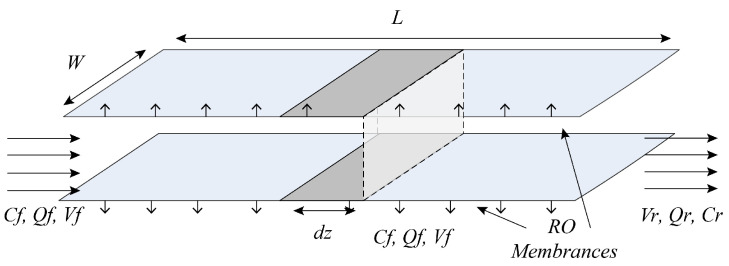
Schematic diagram of the inside of the feeding channel of the spiral-wound RO modules.

The internal channels of the RO membrane element are shown in Figure 2. For the entire high-pressure RO process of the first stage and three stages, a performance model of the RO process can be established based on diffusion permeation and film theory, as well as the law of mass conservation, and combined with empirical equations. The model equation considers the influence of temperature on the diffusion transfer process, and its main equations are shown in Equations (1)–(16).

(1) $$Q_{p}=Q_{f}-Q_{r}$$

(2) $$Q_{f}C_{f}=Q_{p}C_{p}+Q_{r}C_{r}$$

(3) $$Q_{p}=n_{l}w\int_{0}^{L}J_{v}dz=W\int_{0}^{L}J_{v}dz$$

(4) $$J_{v(z)}=A_{w}\left(\Delta P_{b(z)}-\Delta \pi_{\left(z\right)}\right)$$

(5) $$J_{s(z)}=B_{s}\left(C_{m(z)}-C_{p(z)}\right)$$

(6) $$\Delta P_{b(z)}=P_{b(z)}-P_{p(z)}$$

(7) $$\Delta \pi_{\left(z\right)}=RT\left(C_{m(z)}-C_{p(z)}\right)$$

(8) $$\phi_{\left(z\right)}=\frac{C_{m(z)}-C_{p(z)}}{C_{b(z)}-C_{p(z)}}=\exp\left(\frac{J_{v(z)}}{k_{c(z)}}\right)$$

(9) $$A_{w}=A_{w0}\exp\left[\alpha_{1}\frac{T-273}{273}-\alpha_{2}\left(P_{f}-P_{d(z)}\right)\right]$$

(10) $$B_{s}=B_{s0}\exp\left(\beta_{1}\frac{T-273}{273}\right)$$

(11) $$Sh=\frac{k_{c(z)}d_{e}}{D_{AB(z)}}=k_{1}Re^{0.875}Sc^{0.25}$$

(12) $$Re=\frac{\rho_{\left(z\right)}V_{b(z)}d_{e}}{\mu_{\left(z\right)}}$$

(13) $$Sc=\frac{u_{\left(z\right)}}{\rho_{\left(z\right)}D_{AB(z)}}$$

(14) $$D_{AB(z)}=\gamma_{1}\cdot \exp\left(\gamma_{2}C_{b(z)}-\frac{2513}{273.15+T}\right)$$

(15) $$\rho_{\left(z\right)}=\omega_{1}\cdot M+\sqrt{\omega_{2}\cdot M^{2}+\omega_{3}\cdot M\cdot C_{b(z)}}$$

(16) $$\mu_{\left(z\right)}=\eta_{1}\exp(\eta_{2}C_{b(z)}-\frac{1965}{273.15+T})$$

The inter-segment relationships of the first-stage and three-stage high-pressure roll-type RO process can be represented by Equations (17)–(22):

(17) $$Q_{f2}=\frac{Q_{r1}\cdot NP1}{NP2}$$

(18) $$Q_{f3}=\frac{Q_{r2}\cdot NP2}{NP3}$$

(19) $$C_{f2}=C_{r1}$$

(20) $$C_{f3}=C_{r2}$$

(21) $$P_{f2}=P_{r1}+P_{boost}$$

(22) $$P_{f3}=P_{r2}$$

Performance parameters of the RO process:

RO process water recovery rate:

(23) $$R_{ec-RO}=\frac{Q_{p1}+Q_{p2}+Q_{p3}}{Q_{f}}\times 100\%$$

RO process salt rejection rate:

(24) $$R_{y-RO}=(1-\frac{Q_{p1}\cdot C_{p1}+Q_{p2}\cdot C_{p2}+Q_{p3}\cdot C_{p3}}{Q_{f}\cdot C_{f}})\times 100\%$$

### 2.2. Mechanism Model of the MVR Process

**Figure 3:**
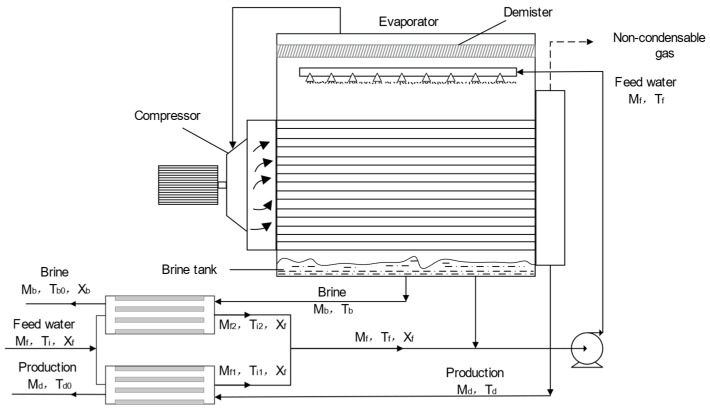
Process flow diagram of the MVR process.

The process flow diagram of the MVR process is shown in Figure 3. The concentrated brine generated by the RO process serves as the feed water for the MVR process. The feed water undergoes sufficient heat exchange between the preheater and the concentrated brine and the condensate generated by the evaporator to increase the temperature of the feed water. The feed water then reaches the evaporator through a circulating pump and is evenly sprayed on the hot pipeline of the evaporator through a nozzle, causing the feed water to be heated. The resulting solution vapor flows through the mist eliminator into a steam compressor, which compresses the steam into high-temperature and high-pressure superheated gas. The compressed gas enters the pipeline of the evaporator and then heats the sprayed feed water. The feed brine then becomes a higher-concentration concentrated brine. At the same time, accompanied by the generation of steam, the steam continues to circulate through the compressor, causing the evaporator and compressor process to reach an equilibrium state. 

**Figure 4:**
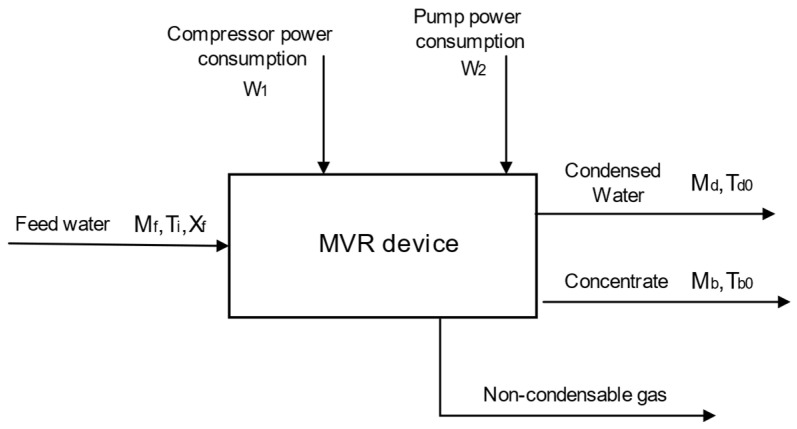
Energy balance diagram of the MVR process.

The establishment of the MVR process mechanism model relies on the energy conservation and mass balance of the entire system and various process. The energy balance diagram of the MVR process is shown in Figure 4. The energy consumption of the MVR process mainly comes from the circulating pump and compressor. To facilitate theoretical analysis, the MVR process model is simplified as follows [27,34,35]:

(1) It is assumed that the salt concentration of the steam and condensate water after evaporation of the saline water is 0%.
(2) It is assumed that the energy losses of the pipelines and pump process in the preheater, evaporator, and evaporator are negligible.
(3) It is assumed that the condensed water obtained after steam passes through the pipeline is in a liquid state.
(4) This study only considers the system in an equilibrium state for mechanistic modeling of the MVR process.

Mass balance of the solution:

(25) $$M_{f}=M_{b}+M_{d}$$

Mass balance of the solute:

(26) $$M_{f}X_{f}=M_{b}X_{b}+M_{d}X_{d}$$

In the preheater:

(27) $$Q_{HEX1}=M_{d}C_{pd}(T_{d}-T_{d0})=M_{f1}C_{pf}(T_{i1}-T_{i})$$

(28) $$Q_{HEX2}=M_{b}C_{pb}(T_{b}-T_{b0})=M_{f2}C_{pf}(T_{i2}-T_{i})$$

In the evaporator:

(29) $$Q_{out}=M_{d}\lambda_{vp}+M_{f}\cdot C_{pf}\left(T_{b}-T_{f}\right)$$

(30) $$Q_{in}=M_{d}\lambda_{d}+M_{d}\cdot C_{pv}\left(T_{s}-T_{d}\right)$$

(31) $$Q_{in}=Q_{out}$$

(32) $$T_{vp}=T_{b}-BPE$$

(33) $$BPE=f\cdot {\Delta_{0}}^{\prime }$$

(34) $$f=0.0162\times \frac{T_{vp}{\;}^{2}}{r}$$

(35) $${\Delta_{0}}^{\prime }=69.8\times C_{b}{\;}^{3}-8.15\times C_{b}{\;}^{2}+5.19\times C_{b}$$

(36) $$T_{vp}=T_{v}$$

(37) $$\Delta T=T_{d}-T_{b}$$

(38) $$Q_{e}=U_{e}\cdot A_{e}\cdot LTMD$$

(39) $$U_{e}=1.9695+1.2057\times 10^{-2}\times T_{b}-8.5989\times 10^{-5}\times T_{b}^{2}+2.565\times 10^{-7}\times T_{b}^{3}$$

### 2.3. Model Validation of the RO Process

To ensure the correctness of model establishment, model validation is necessary. This study focuses on the wastewater treatment process of a coal-fired power plant in Inner Mongolia, comparing the model calculation results with the actual data from the plant. Table 1 and Table 2 list the membrane element parameters and feed water quality parameters used in the RO process.

By simulating and analyzing the model, real-time monitoring and transparent management of the power plant can be achieved, and optimization of the zero-discharge wastewater system can be further realized. Therefore, the correctness of the model is a prerequisite. To validate the model’s accuracy, this paper takes the operating condition data in Table 2 as inputs for the model and Winflows software (version 4.03) [36]. The calculated results of the model are then compared with the simulation results from the Winflows design software to preliminarily verify the model’s correctness. The comparison results are shown in Table 3.

After comparative analysis, it can be observed that the water flowrate errors for the first stage, second stage, and third stage are 7.1%, 12%, and 12%, respectively. The water recovery rate error is 7.6%, and the desalination rate error is 6.06%. Only the water flowrate errors in the second and third stages are relatively large, while the other errors are relatively small. Due to the differences in the design software version, there may be changes in the membrane parameters within the system. However, in the actual operation of the power plant, these errors can be acceptable. Thus, these results initially prove the effectiveness of the model established in this paper.

To further validate the accuracy of the model, a comparison is made between the actual plant data and the model calculation data. Three sets of data under different operating conditions are selected for calculation and comparison. The plant data and the model calculation data are shown in Table 4. From the comparison of the three sets of data in the table, it can be seen that the final water concentration errors for the three sets of data are 0.26 kg/m^3^, 0.28 kg/m^3^, and 0.37 kg/m^3^, respectively. There are many reasons for these errors, such as measurement errors of sensors. However, all of the errors are within an acceptable range, thus providing further evidence of the effectiveness of the model.

### 2.4. Model Validation of the MVR Process

In order to ascertain the accuracy of the constructed model for MVR, computational simulations were performed using GAMS software (version 25.1.1) [37]. The model data were compared with the simulated operating conditions described in the study conducted by Helal et al. [38]. The equipment parameters for the MVR process are listed in Table 5, while the specific parameters for the simulated operating conditions can be found in Table 6.

Maintaining consistency with the aforementioned conditions, the comparative results of relevant calculations, in comparison with the data presented in Helal et al.’s [38] study, are shown in Table 7. The errors in the mass flowrates of the concentrated brine and condensate water are 0.004 kg/h and 50.054 kg/h, respectively. Compared with the data in Helal et al.’s [38], the maximum error in the calculated results is 1.01%. The temperature differences observed for the feed water, concentrated brine, condensate water, and superheated steam are 1.788 °C, 0.639 °C, 1.301 °C, and 0.778 °C, respectively, with a maximum error of 3.33%. However, there are relatively larger errors in the temperature increase of the boiling point, the heat transfer coefficient of the evaporator, and the heat transfer area of the evaporator. This is because the MVR model in this study has been simplified and certain conditions were assumed, while the model by Helal et al. [38] takes into account various factors such as spray angle in the evaporator, scaling effects in the evaporator pipes, and incomplete heat transfer in the heat exchanger. These results serve to validate the accuracy of the MVR mechanism model.

### 2.5. Mechanism Modeling and Economic Model of RO-MVR Joint System 

**Figure 5:**
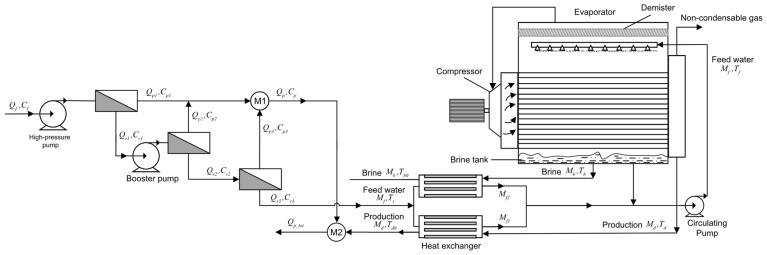
Process flow diagram of the RO-MVR joint system.

In order to study the optimization of the operation of the RO-MVR joint system, a mechanism model of the RO-MVR joint system is established based on the first-stage and three-stage high-pressure RO and MVR process models established above. The concentrated brine generated by the first-stage and third-stage RO process is used as feed water for the MVR process, and the process flow diagram is shown in Figure 5.

The connection model between the RO process and the MVR process is as follows:

(40) $$M_{f}=Q_{r3}\cdot 1000$$

(41) $$X_{f}=C_{r3}/10$$

Freshwater produced by the RO-MVR system:

(42) $$Q_{p\_tot}=Q_{p\_RO}+M_{d}$$

Brine water produced by the RO-MVR system:

(43) $$Q_{b\_tot}=M_{b}$$

To achieve optimization of the operation of the joint system, it is first necessary to establish a correct economic model for the operating costs of the coal electricity wastewater joint system, and then optimize the solution through the GAMS platform to obtain the optimal strategy for optimizing the operation of the coal electricity wastewater membrane method. This article studies the operating costs of a 24-h RO-MVR system, so the RO cleaning and maintenance costs can be ignored. Moreover, the MVR process is in a stable operating state, so the cost of the MVR process mainly consists of the costs of the compressor and circulating pump. The operating cost of the RO-MVR joint system for treating coal-fired wastewater through the membrane method mainly includes the following parts:

(1) Chemical addition cost ($OC_{CH}$);
(2) Water intake energy consumption cost ($OC_{IP}$);
(3) Energy consumption cost of the RO unit operation ($OC_{EN}$);
(4) The cost of replacing the RO membrane ($OC_{ME}$);
(5) Maintenance cost ($OC_{MN}$);
(6) Labor cost ($OC_{LB}$);
(7) The energy consumption of the MVR process’s circulating pump ($OC_{\mathrm{cir}}$);
(8) The energy consumption of the MVR process’s compressor ($OC_{\mathrm{com}}$).

The specific operating costs for each part are as follows:

(44) $$OC_{CH}=kQ_{f}$$

(45) $$OC_{IP}=\frac{P_{0}\cdot Q_{f}\cdot P_{elc}}{c}\times PLF$$

(46) $$OC_{EN}=[\frac{P_{f}Q_{f}/(\varepsilon_{p}\cdot \varepsilon_{VFD})+P_{boost}\cdot Q_{f2}/\varepsilon_{bp}}{Q_{p}}]\times P_{elc}$$

(47) $$OC_{ME}=\mathrm{Pr}i_{ME}\cdot NM\cdot \zeta_{re}/360$$

(48) $$OC_{MN}=\sigma OC_{RO}$$

(49) $$OC_{LB}=\mathrm{Pr}i_{LB}\cdot N_{LB}$$

(50) $$OC_{cir}=M_{f}\cdot \rho \cdot P_{elc}/\eta_{p}$$

(51) $$OC_{com}=(H_{s}-Hv)\cdot 0.000277\cdot \varepsilon \cdot P_{elc}/\eta_{com}$$

Total operating expenses:

(52) $$OC=OC_{CH}+OC_{IP}+OC_{EN}+OC_{ME}+OC_{MN}+OC_{LB}+OC_{cir}+OC_{com}$$

Unit water production cost:

(53) $$OC_{\mathrm{unit}}=\frac{OC}{Q_{P\_tot}}$$

In order to quantitatively analyze the RO-MVR joint system, the equipment parameters for RO and MVR are set according to Table 1 and Table 5, respectively, and the operating conditions and cost parameters of the MVR device are shown in Table 8.

### 2.6. Operational Optimization Based on the Goal of Minimizing Water Production Unit Price

Based on the optimization proposition of the lowest unit price of water production per unit, under the parameter setting of the RO-MVR joint system for coal power wastewater, the data of three typical operating conditions are given based on the actual operation situation of the factory, and optimized and solved through the GAMS platform. Before optimization, the feed flowrate, operating pressure, and booster pump pressure of the fixed RO-MVR joint system were fixed. After optimization, based on considerations of the safety and actual operation of the RO-MVR system, the upper and lower bounds of the feed flowrate, operating pressure, and booster pump pressure were given. Based on the optimization proposition of the lowest water production unit price, the scheme was solved and three data results were obtained: before the optimization of the RO-MVR joint system, only the optimization of the RO process, and after the optimization of the RO-MVR joint system.

Case 1: The feed temperature is 15 °C, the feed concentration is 30.0 kg/m^3^, the feed flowrate is 28.8 m^3^/h, the operating pressure is 31.8 bar, and the pressure of the booster pump is 35 bar.

Case 2: The feed temperature is 15 °C, the feed concentration is 23.3 kg/m^3^, the feed flowrate is 28.8 m^3^/h, the operating pressure is 31.8 bar, and the pressure of the booster pump is 35 bar.

Case 3: The feed temperature is 15 °C, the feed concentration is 30.0 kg/m^3^, the feed flowrate is 25.6 m^3^/h, the operating pressure is 31.8 bar, and the pressure of the booster pump is 30 bar.

Objective function:

$$\underset{P_{f},Q_{f},P_{boost},T}{\mathrm{Min}}OC_{unit}$$

Equality constraints:

RO process model (Equations (1)–(24));

MVR mechanism model (Equations (25)–(39));

RO-MVR system connection model (Equations (40)–(43));

RO-MVR operating cost model (Equations (44)–(53)).

Inequality constraints:

$$P_{f}^{L}\leq P_{f}\leq P_{f}^{U}$$

$$P_{\mathrm{boost}}^{L}\leq P_{boost}\leq P_{boost}^{U}$$

$$C_{p,out}\leq C_{\lim\;it}$$

$$V_{f}^{L}\leq V_{f}\leq V_{f}^{U}$$

$$\phi^{L}\leq \phi \leq \phi^{U}$$

$$T_{f}^{L}\leq T_{f}\leq T_{f}^{U}$$

Initial conditions:

$$P_{b}=\left\{\begin{array}{c} P_{f},z=0\\ P_{r},z=L \end{array}\right.$$

$$C_{b}=\left\{\begin{array}{c} C_{f},z=0\\ C_{r},z=L \end{array}\right.$$

$$V=\left\{\begin{array}{c} V_{f}=\frac{Q_{f}}{n_{e}Wh_{sp}},z=0\\ V_{f}=\frac{Q_{r}}{n_{e}Wh_{sp\_r}},z=L \end{array}\right.$$

### 2.7. Operational Optimization Based on the Goal of Minimizing Daily Operating Costs

Based on the operation optimization proposition of the lowest daily operating cost, under the parameter setting of the coal power wastewater RO-MVR joint system, the data for three typical operating conditions are given based on the actual operation situation of the factory, and optimized and solved through the GAMS platform. Before optimization, the feed flowrate, operating pressure, and booster pump pressure of the fixed RO-MVR joint system are fixed. After optimization, based on considerations of the safety and actual operation of the RO-MVR system, the upper and lower bounds of the feed flowrate, operating pressure, and booster pump pressure are given. The optimization proposition with the lowest daily operating cost is solved, and the three results—before the optimization of the RO-MVR joint system, only the optimization of the RO process, and after the optimization of the RO-MVR joint system—are analyzed.

Case 1: The feed temperature is 15 °C, the feed concentration is 30.0 kg/m^3^, and the feed flowrate is 28.8 m^3^/h. The operating pressure is 31.8 bar, and the pressure of the booster pump is 35 bar.

Case 2: The feed temperature is 15 °C, the feed concentration is 23.3 kg/m^3^, and the feed flowrate is 28.8 m^3^/h. The operating pressure is 31.8 bar, and the pressure of the booster pump is 35 bar.

Case 3: The feed temperature is 15 °C, the feed concentration is 30.0 kg/m^3^, and the feed flowrate is 25.6 m^3^/h. The operating pressure is 31.8 bar, and the pressure of the booster pump is 30 bar.

Objective function:

$$\underset{P_{f},Q_{f},P_{boost},T}{Min}OC$$

Equality constraints:

RO process model (Equations (1)–(24));

MVR mechanism model (Equations (25)–(39));

RO-MVR system connection model (Equations (40)–(43));

RO-MVR operating cost model (Equations (44)–(53)).

Inequality constraints:

$$P_{f}^{L}\leq P_{f}\leq P_{f}^{U}$$

$$P_{\mathrm{boost}}^{L}\leq P_{boost}\leq P_{boost}^{U}$$

$$C_{p,out}\leq C_{\lim\;it}$$

$$V_{f}^{L}\leq V_{f}\leq V_{f}^{U}$$

$$\phi^{L}\leq \phi \leq \phi^{U}$$

$$T_{f}^{L}\leq T_{f}\leq T_{f}^{U}$$

Initial conditions:

$$P_{b}=\left\{\begin{array}{c} P_{f},z=0\\ P_{r},z=L \end{array}\right.$$

$$C_{b}=\left\{\begin{array}{c} C_{f},z=0\\ C_{r},z=L \end{array}\right.$$

$$V=\left\{\begin{array}{c} V_{f}=\frac{Q_{f}}{n_{e}Wh_{sp}},z=0\\ V_{f}=\frac{Q_{r}}{n_{e}Wh_{sp\_r}},z=L \end{array}\right.$$

## 3. Results and Discussion

### 3.1. Operational Optimization Based on the Goal of Minimizing the Water Production Per-Unit Price

Based on the optimization proposition of the lowest price of water production per unit, under the parameter setting of the RO-MVR joint system for coal power wastewater, the data for three typical operating conditions are given based on the actual operation situation of the factory, and optimized and solved through the GAMS platform. The results of this are shown in Table 9.

Case 1 was optimized and solved; the optimal feeding conditions were obtained: the feed water flowrate was 32.6 m^3^/h, the feed water pressure was 39.6 bar, and the pressure of the booster pump was 25.9 bar. As shown in Table 9, it is clear from the optimization solution for Case 1 that when only the RO process was optimized, the salt rejection rate of the RO-MVR system increased by 0.74%, the water recovery rate increased by 6.1%, the water concentration decreased by 0.35 kg/m^3^, and the unit cost of water production decreased by 0.82 CNY/m^3^. However, after the joint optimization of the RO-MVR system, although the system’s water recovery rate did not change much, the salt rejection rate, the water concentration, and the unit price of the joint system improved further than before: the salt rejection rate increased by 1.87%, and the water concentration dropped from the original 1.24 kg/m^3^ to 0.53 kg/m^3^, a decrease of 57.26%. This is 29.03% lower than the water concentration when only the RO unit was optimized, resulting in a significant improvement in water quality. Meanwhile, the unit cost of water production also decreased by 1.21 CNY/m^3^ after optimization: a decrease of 26.65%, and a further 8.59% reduction from the original basis, achieving the goal of further reducing the unit cost of water production.

Case 2 was optimized and solved; the optimal feeding conditions were obtained: the feed water flowrate was 36.8 m^3^/h, the feed water pressure was 33.1 bar, and the pressure of the booster pump was 29.6 bar. With optimization carried out on the RO process only, compared to before optimization, the salt rejection of the RO-MVR system increased by 1.37%, the water recovery rate increased by 3.14%, the concentration of product water decreased by 0.41 kg/m^3^, and the cost per cubic meter of product water decreased by 0.76 CNY/m^3^. After combined optimization of the RO-MVR system, although the water recovery rate decreased by1.68%, the salt rejection increased by 2.7%, and the water concentration of the system decreased by 0.74 kg/m^3^ compared to before optimization, reducing by 62.18%, which is 27.73% lower than the water concentration after optimization of the RO process alone. Similarly, the cost of water production of the system after combined optimization decreased by 1.09 CNY/m^3^: a decrease of 25.65%, further reducing by 7.77% on the basis of the former. Therefore, the combined optimization not only reduced the cost of water production and lessened the costs of operation, but also improved the quality and water recovery rate of the product water, thereby enhancing the cost-effectiveness of water production.

Case 3 was optimized and solved; the optimal feeding conditions were obtained: the feed water flowrate was 32.6 m^3^/h, the feed water pressure was 40.1 bar, and the pressure of the booster pump was 25.2 bar. In the case of only optimizing the RO process, compared to before optimization, the salt rejection of the RO-MVR system increased by 0.84%, the water recovery rate increased by 6.19%, and the water production concentration decreased by 0.39 kg/m^3^, with the unit price of water production being reduced by 0.54 CNY/m^3^. After joint optimization of the RO-MVR system, the performance of the system was improved to a certain extent, with a 2.19% increase in the salt rejection rate and a 1.22% increase in the water recovery rate. The water production concentration of the system decreased by 0.82 kg/m^3^ compared to before optimization, a reduction of 60.74%, with a reduction of 31.85% on the basis of the individual RO process optimization. Similarly, the per-unit price of water production in the jointly optimized system was reduced by 0.93 CNY/m^3^: a decrease of 21.88%, and a decrease of 9.17% on the basis of the former. Therefore, this joint optimization not only reduced the per-unit price of water production and lessened the operating costs, but also improved the water quality and cost-effectiveness of water production.

From an analysis of the above results, it can be seen that compared to the operation optimization only for the RO process, the combined optimization based on the lowest per-unit cost of water production in the RO-MVR joint system carried out in this study can further reduce the water production cost and improve the water production quality and water recovery rate of the system to a certain extent. Among these, the operating cost of water production in the system was reduced by more than one-fifth after combined optimization.

### 3.2. Operational Optimization Based on the Goal of Minimizing Daily Operating Costs

In terms of operating costs, labor costs, daily maintenance costs, and membrane replacement costs, these are not related to the flowrate, so these values are fixed before and after optimization. However, changes in other costs can cause changes in the proportion of these values. Correspondingly, pre-treatment energy costs, chemical costs, and operating energy costs are related to the system’s feed parameters, so these values change before and after optimization.

Table 10 show the comparative results of three scenarios under Case 1 conditions: before the joint optimization of the RO-MVR system, with only the optimization of the RO process, and after the joint optimization of the RO-MVR system. In Case 1, the overall operating cost of a single system before joint optimization was 2513.90 CNY/day, with the sum of the system’s operating energy consumption and MVR compressor energy consumption accounting for 54.81% of the total cost. The maintenance cost of a single system is 120 CNY/day, which is less than 5% of the total operating cost, and there is not much room for cost savings. Comparing the total operating costs before and after system optimization, it can be seen that the total operating costs of the system were significantly decreased after joint optimization. The optimal daily operating cost of the entire process decreased to CNY 18,250.4, a decrease of over 27%, which is CNY 6725.6 less than under the condition of only optimizing the RO process. Among them, the operating energy consumption cost of the entire process was decreased from 6904.1 CNY/day to 2341.0 CNY/day, a decrease of up to 66%. In the total cost of the entire process, excluding membrane replacement, maintenance, and labor costs, the total cost was reduced from CNY 17,605.7 to CNY 10,717.1, and the system saved nearly two-fifths of the cost. 

Table 11 show the comparative results of three scenarios under Case 2 conditions: before the joint optimization of the RO-MVR system, with only the optimization of the RO process, and after the joint optimization of the RO-MVR system. Under the operating conditions of Case 2, the overall operating cost of the entire process before joint optimization was 24,787.3 CNY/day, with the compressor energy consumption and operating energy consumption accounting for the highest proportion, accounting for 28.44% and 27.85%, respectively, followed by labor costs. Comparing the total daily operating costs of the system before and after joint optimization, it can be found that the total operating costs of the system significantly decreased after optimization. The daily operating costs of the entire process were decreased to CNY 18,045.1, a decrease of 27.2%. Excluding fixed costs (including labor costs, maintenance costs, and membrane replacement costs), the entire process cost was decreased from CNY 17,254.0 to CNY 10,511.8; compared to the situation where only the RO process was optimized, it also reduced by CNY 6434.6, achieving the goal of energy saving and money saving.

Table 12 show the comparative results of three scenarios under Case 3 conditions: before the joint optimization of the RO-MVR system, with only the optimization of the RO process, and after the joint optimization of the RO-MVR system. Before joint optimization, the overall operating cost of a single system was 2333.77 CNY/day. The energy consumption of the MVR compressor (*OC_com_*) and the operating energy consumption of the system (*OC_EN_*) still represented the top two most expensive, accounting for 28.58% and 24.26% of the total cost, respectively. Comparing the total operating costs before and after joint optimization of the system, it can be seen that the total operating costs of the system were significantly decreased after joint optimization. The optimal daily operating cost of the entire process was decreased to CNY 18,184.2, a decrease of more than 22%, saving 15.44% of the cost compared to only optimizing the RO process. In the total cost of the entire process, excluding membrane replacement, system maintenance, and labor costs, the total cost decreased from CNY 15,894.4 to CNY 10,650.09, achieving the goal of reducing operating costs.

**Figure 6:**
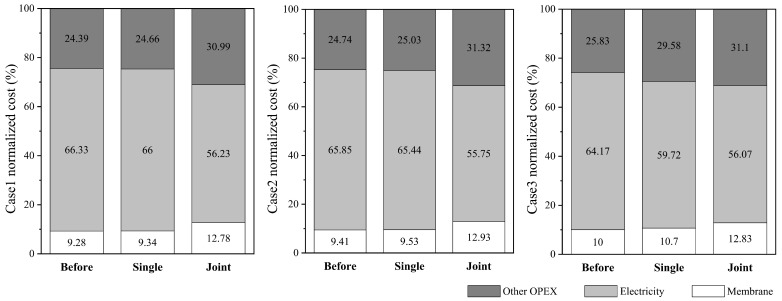
Normalized component costs for Cases 1–3.

Figure 6 provides the normalized costs for Cases 1–3: the membrane cost (*OC_ME_*), the electricity cost (*OC_EN_*, *OC_IP_*, *OC_cir_*, *OC_com_*) and other operating costs (*OC_CH_*, *OC_LB_*, *OC_MN_*). Among them, the electricity cost is the largest component of the total costs, accounting for 55–66% of the total cost before and after optimization, followed by other operating costs, and the membrane cost accounts for the smallest proportion, only 9–13% of the total cost. After optimizing the joint system, the proportion of electricity costs decreased by 10.1%, 10.1%, and 8.1%, respectively. Due to the fixed labor costs, daily maintenance costs, and membrane replacement costs, the proportion of optimized membrane costs and other operating costs was increased, providing a direction for us to further reduce wastewater treatment costs in the future.

In summary, in the optimization process aimed at the lowest daily operating cost, under typical operating conditions of the three types of coal-fired power plants, the system operating energy cost (*OC_EN_*) accounted for a relatively high proportion of the total system cost before joint optimization, while the proportion that the system operating energy consumption cost accounted for in the total cost decreased by more than 10% after joint optimization. Meanwhile, compared to the total operating cost of only optimizing the RO process, the total operating cost of the RO-MVR joint system optimization was lower, and the system could save up to 27% of the cost.

## 4. Conclusions

The production of coal-fired power plants generates a large amount of high-salinity wastewater, and the recycling and utilization of high-salinity wastewater is an important way to solve water resource shortages and protect the environment. This article takes the saline wastewater treatment process of a coal power plant in Inner Mongolia as the background, and based on the characteristics of the coal power wastewater process, establishes the mechanism models and operating cost models of a three-stage, high-pressure, roll-type RO device and MVR device, respectively. On this basis, an operation optimization of the RO-MVR joint system was carried out. Firstly, a mechanism model of the RO process was established based on diffusion permeation, film theory, and mass conservation. Secondly, a mechanism model of the MVR process was established based on the principles of mass conservation and energy conservation. Then, the correctness of the established RO model and MVR model was verified. Next, considering the economic cost issue, an economic model of the RO-MVR joint system was established. The optimization objective of the RO-MVR joint system was to minimize the water production unit price, and three different operating conditions were studied for optimization. The results showed that the optimized water production price per unit could be reduced to 3.33 CNY/m^3^, 3.16 CNY/m^3^, and 3.32 CNY/m^3^. Compared to before optimization, this was decreased by 25.65%, 26.65%, and 21.88%, respectively. In addition, the optimized production water concentration was also reduced, fully demonstrating the effectiveness of operational optimization. Then, this article took the minimum daily operating cost of the RO-MVR joint system as the optimization objective and studied the optimization situation under three different operating conditions. The results show that the optimized daily operating cost of the entire device was reduced by 6888.6 CNY/day, 6742.2 CNY/day, and 5153.5 CNY/day, respectively. And the operating cost was reduced by 27%, 27%, and 22%, achieving the goal of reducing wastewater treatment costs and providing assistance for coal-fired power plants to reduce the costs of wastewater treatment. In future work, we will focus on case studies of optimal control in wastewater treatment of coal-fired power plants.

## Figures and Tables

**Table 1:** Parameters of high-pressure RO membrane elements.

Membrane Property	Unit	Value
RO membrane model	—	INDUSTRIAL RO7 8040F35
Effective area	m^2^	30.7
Effective membrane leaf length	m	0.827
Effective membrane leaf width	m	0.64
Inlet mesh thickness	mm	0.889
Average flow	m^3^/d	39.7
Maximum operating pressure	bar	120
Maximum operative temperature	°C	50
Pump efficiency	—	0.75

**Table 2:** Feed water quality parameters of the high-pressure RO process.

Property	Result (mg/L)	Property	Result
K^+^	226.00	Total alkalinity	479.7 mmol/L
Na^+^	6149.00	Non-carbonate hardness	478.1 mmol/L
1/2 Ca^2+^	723.45	Carbonate hardness	1.6 mmol/L
1/2 Mg^2+^	5389.74	Total silica	122.7 mg/L
NH_4_^+^	118.00	Inactive silicon	41.5 mg/L
1/2 Sr^2+^	3.50	TDS	45,082 mg/L
1/2 Ba^2+^	0.10	SS	62 mg/L
1/3 Fe^3+^	0.21	Phosphorus	1.2 mg/L
Total cations	12,610.00	Ammonia nitrogen	91.5 mg/L
Cl^−^	15,410.00	Turbidity	21.7 NTU
1/2 SO_4_^2−^	15,931.00	PH (25 °C)	6.25
HCO_3_^−^	97.63	Water temperature	15 °C
NO_3_^−^	732.00	Water pressure	31.8 bar
F^−^	4.60	Water flowrate	28.8 m^3^/h
Total anions	32,175.23	Water concentration	23,300 mg/L

**Table 3:** Calculation results of the model and the simulation results of Winflows.

Data Sources	$Q_{p1}$ (m^3^/h)	$Q_{p2}$ (m^3^/h)	$Q_{p3}$ (m^3^/h)	$R_{ec-RO}$ (%)	$R_{y-RO}$ (%)
Winflows data (δ)	11.59	7.13	1.89	71.56	90.81
Model data (θ)	10.762	6.252	2.121	66.13	96.31
Error comparison	0.071	0.12	0.12	0.076	6.06

$Q_{p1}$, $Q_{p2}$, and $Q_{p3}$ represent the production water flow of the first, second, and third stages of the RO process, respectively. error=|δ−θ|/δ.

**Table 4:** Comparison between factory data and model data for the RO membrane.

Parameters	Data 1		Data 2		Data 3	
	Factory	Model	Factory	Model	Factory	Model
*Q_f_* (m^3^/h)	18.92	18.92	19.29	19.29	23.71	23.71
*P_f_* (bar)	33.73	33.73	34.83	34.83	33.85	33.85
*C_f_* (kg/m^3^)	26.58	26.58	26.54	26.54	26.09	26.09
*Q_p1_* (m^3^/h)	—	6.90	—	7.28	—	8.24
*Q_p2_* (m^3^/h)	—	3.98	—	3.97	—	4.98
*Q_p3_* (m^3^/h)	—	1.34	—	1.33	—	1.76
*C_p1_* (kg/m^3^)	—	1.46	—	1.42	—	1.16
*C_p2_* (kg/m^3^)	—	2.08	—	2.12	—	1.55
*C_p3_* (kg/m^3^)	—	7.64	—	7.80	—	5.75
*C_p_last_* (kg/m^3^)	2.59	2.33	2.59	2.31	2.20	1.83

**Table 5:** MVR equipment parameters.

Equipment Parameters	Value
Outer diameter of evaporator tube (m), *D_i_*	0.03
Compression ratio of the compressor	1.22
Mechanical efficiency	0.75
Temperature difference of evaporator (°C), Δ*T*	5
Initial temperature of the solution (°C), *T_i_*	17

**Table 6:** Parameters of the simulated operating conditions.

Parameters	Value
Feed brine concentration (%), *X_f_*	4.5
Concentrated brine concentration (%), *X_b_*	8.03583
Condensate concentration (%), *X_d_*	0
Feed flowrate (kg/h), *M_f_*	11,196.65
Steam temperature (°C), *T_v_*	55
Temperature of concentrated brine after passing through the preheater (°C), *T_b0_*	23

**Table 7:** Comparative results of MVR model calculations.

Parameters	Model Data	Helal’s Data
Concentrated brine mass flowrate (kg/h), *M_b_*	6270.034	6270.03
Condensate mass flowrate (kg/h), *M_d_*	4926.616	4976.67
Temperature of feed water after passing through the preheater (°C), *T_f_*	51.632	53.42
Temperature of concentrated brine (°C), *T_b_*	55.301	55.94
Temperature of condensate water (°C), *T_d_*	60.301 g	59
Temperature of superheated steam (°C), *T_s_*	71.332	72.11
Rise in boiling point (°C), *BPE*	0.301	0.94
Heat transfer coefficient of evaporator (kw/m^2^/K), *U_e_*	2.417	3.94
Evaporator heat transfer area (m^2^), *A_e_*	7.934	2.2

**Table 8:** Operating conditions of MVR equipment and cost parameters of the joint system.

Parameters	Value
Concentrated brine concentration (%), *X_b_*	20
Steam temperature (°C), *T_v_*	70
Temperature of concentrated brine after passing through the preheater (°C), *T_b0_*	25
Electricity price (CNY/kw·h), *P_elc_*	0.7
Unit price of RO membrane elements (CNY), *Pri_ME_*	7000
Cost per worker (CNY/day), *Pri_LB_*	400
Replacement rate of membrane components, *ξ_re_*	0.25

**Table 9:** Performance comparison results before and after optimization of Cases 1–3.

Item	Case 1			Case 2			Case 3		
	Before	Single	Joint	Before	Single	Joint	Before	Single	Joint
Salt rejection rate (%)	96.70	97.44	98.57	95.70	97.07	98.40	96.38	97.22	98.57
Water recovery rate (%)	80.14	86.26	81.33	84.32	87.46	82.64	80.14	86.33	81.36
Water production concentration (kg/m^3^)	1.24	0.89	0.53	1.19	0.78	0.45	1.35	0.96	0.53
Unit price of water production (CNY/m^3^)	4.54	3.72	3.33	4.25	3.49	3.16	4.74	3.71	3.32

Before: before joint optimization; Single: single RO optimized; Joint: joint optimized.

**Table 10:** Composition of operating costs before and after Case 1 optimization.

Items	Before Joint Optimization			Single RO Optimized			Joint Optimized		
	Single (CNY)	Entire (CNY)	Ratio (%)	Single (CNY)	Entire (CNY)	Ratio (%)	Single (CNY)	Entire (CNY)	Ratio (%)
*OC_CH_*	93.31	933.1	3.71	95.98	959.8	3.84	45.52	455.2	2.49
*OC_EN_*	690.41	6904.1	27.46	722.50	7225.0	28.93	234.10	2341.0	12.83
*OC_IP_*	71.12	711.2	2.83	73.22	732.2	2.93	34.70	347.0	1.90
*OC_LB_*	400.00	4000.0	15.91	400.00	4000.0	16.02	400.00	4000.0	21.92
*OC_ME_*	233.33	2333.3	9.28	233.33	2333.3	9.34	233.33	2333.3	12.78
*OC_MN_*	120.00	1200.0	4.77	120.00	1200.0	4.80	120.00	1200.0	6.58
*OC_cir_*	218.11	2181.1	8.68	224.01	2240.1	8.97	128.62	1286.2	7.05
*OC_com_*	687.62	6876.2	27.35	628.56	6285.6	25.17	628.77	6287.7	34.45
*OC*	2513.90	25,139.0	100	2497.60	24,976.0	100	1825.04	18,250.4	100

**Table 11:** Composition of operating costs before and after Case 2 optimization.

Items	Before Joint Optimization			Single RO Optimized			Joint Optimized		
	Single (CNY)	Entire (CNY)	Ratio (%)	Single (CNY)	Entire (CNY)	Ratio (%)	Single (CNY)	Entire (CNY)	Ratio (%)
*OC_CH_*	93.31	933.1	3.76	92.80	928.0	3.79	45.18	451.8	2.50
*OC_EN_*	690.22	6902.2	27.85	591.24	5912.4	24.15	221.88	2218.8	12.30
*OC_IP_*	71.12	711.2	2.87	70.78	707.8	2.89	34.40	344.0	1.91
*OC_LB_*	400.00	4000.0	16.14	400.00	4000.0	16.34	400.00	4000.0	22.17
*OC_ME_*	233.33	2333.3	9.41	233.33	2333.3	9.53	233.33	2333.3	12.93
*OC_MN_*	120.00	1200.0	4.84	120.00	1200.0	4.90	120.00	1200.0	6.65
*OC_cir_*	165.91	1659.1	6.69	155.80	1558.0	6.36	121.22	1212.2	6.72
*OC_com_*	704.84	7048.4	28.44	784.03	7840.3	32.03	628.50	6285.0	34.83
*OC*	2478.73	24,787.3	100	2447.98	24,479.8	100	1804.51	18,045.1	100

**Table 12:** Composition of operating costs before and after Case 3 optimization.

Items	Before Joint Optimization			Single RO Optimized			Joint Optimized		
	Single (CNY)	Entire (CNY)	Ratio (%)	Single (CNY)	Entire (CNY)	Ratio (%)	Single (CNY)	Entire (CNY)	Ratio (%)
*OC_CH_*	82.94	829.4	3.55	76.63	766.3	3.51	45.51	455.1	2.50
*OC_EN_*	566.15	5661.5	24.26	439.73	4397.3	20.16	230.31	2303.1	12.67
*OC_IP_*	63.25	632.5	2.71	58.43	584.3	2.68	34.70	347.0	1.91
*OC_LB_*	400.00	4000.0	17.14	400.00	4000.0	18.34	400.00	4000.0	22.00
*OC_ME_*	233.33	2333.3	10.00	233.33	2333.3	10.70	233.33	2333.3	12.83
*OC_MN_*	120.00	1200.0	5.14	120.00	1200.0	5.50	120.00	1200.0	6.60
*OC_cir_*	201.06	2010.6	8.62	168.56	1685.6	7.73	131.12	1311.2	7.21
*OC_com_*	667.04	6670.4	28.58	684.15	6841.5	31.37	623.45	6234.5	34.29
*OC*	2333.77	23,337.7	100	2180.83	21,808.3	100	1818.42	18,184.2	100

## Data Availability

Data are contained within the article.

## Abbreviations

*A_e_*	heat exchange area of the evaporator (m^2^)
*A_w_*	membrane water permeability (m·s^−1^·Pa^−1^)
*A_w0_*	intrinsic membrane water permeability (m·s^−1^·Pa^−1^)
*BPE*	boiling point elevation (°C)
*B_s_*	membrane TDS permeability (m/s)
*B_s0_*	intrinsic membrane TDS permeability (m/s)
*C_b_*	brine concentration (kg/m^3^)
*C_f_*	feed salt concentration of the RO unit (kg/m^3^)
*C_m_*	saltwater concentration at membrane surface (kg/m^3^)
*C_p_*	permeate concentration of the RO unit (kg/m^3^)
*C_pd_*, *C_pf_*, *C_Pb_*, *C_pv_*	specific heat capacity (kJ/(kg·°C))
*C_r_*	brine concentration of the RO unit (kg/m^3^)
*d_e_*	hydraulic diameter of the feed spacer (m)
*f*	correction factor of MVR unit
*H*	enthalpy (kJ/kg)
*J_s_*	solute flux (kg/m^2^·s)
*J_v_*	solvent flux (kg/m^2^·s)
*K_c_*	mass transfer coefficient
*L*	effective length of the membrane assembly (m)
*M_b_*	brine mass flowrate of the MVR unit (kg/h)
*M_d_*	condensed steam mass flowrate of the MVR unit (kg/h)
*M_f_*	feed mass flowrate of the MVR unit (kg/h)
*n_1_*	number of leaves of the membrane assembly
*NM*	total number of membrane elements in the RO unit
*NP*1	number of pressure vessels in the first stage of the RO unit
*NP*2	number of pressure vessels in the second stage of the RO unit
*NP*3	number of pressure vessels in the third stage of the RO unit
*OC_CH_*	chemical addition cost (CNY)
*OC_cir_*	energy consumption of the MVR unit’s circulating pump (CNY)
*OC_com_*	energy consumption of the MVR unit’s compressor (CNY)
*OC_EN_*	energy consumption cost of the RO unit’s operation (CNY)
*OC_IP_*	water intake energy consumption cost (CNY)
*OC_LB_*	labor cost (CNY)
*OC_ME_*	cost of replacing the RO membrane (CNY)
*OC_MN_*	maintenance cost (CNY)
*P*_0_	the outlet pressure of the water intake pump (bar)
*P_b_*	brine pressure (bar)
*P_boost_*	pressure of the booster pump (bar)
*P_elc_*	electricity price (CNY)
*P_f_*	feed pressure of the RO unit (bar)
*PLE*	load factor
*P_p_*	permeate pressure (bar)
*Pri_ME_*	unit price of RO membrane elements (CNY)
*Q_e_*	heat transfer in the evaporator (KW)
*Q_f_*	feed flowrate of the RO unit (m^3^/h)
*Q_p_*	permeate flowrate of the RO unit (m^3^/h)
*Q_r_*	brine flowrate of the RO unit (m^3^/h)
*R*	gas constant
*R_e_*	Reynolds number
*S_c_*	Schmidt number
*T_b_*	temperature of the concentrated saltwater at the outlet of the evaporator (°C)
*Td*	temperature of the condensed steam (°C)
*T_f_*	temperature after passing through the heat exchanger (°C)
*T_i_*	temperature of the feed water (°C)
*T_s_*	temperature of superheated steam (°C)
*T_v_*	temperature above the demister (°C)
*T_vp_*	temperature below the demister (°C)
*U_e_*	overall heat transfer coefficient (kw/m^2^/K)
*V_b_*	flow velocity inside the channel (kg/h)
*w*	width of the membrane assembly (m)
*W*	total width of the membrane assembly (m)
*X_b_*	mass fraction concentrations of the brine (%)
*X_d_*	mass fraction concentrations of the condensed steam (%)
*X_f_*	mass fraction concentrations of the feed (%)
*z*	position along the membrane channel
*α*_1_, *α*_2_, *β*_1_	transport parameters
*γ*_1_, *γ*_2_	coefficients in the equation
Δ_0_’	boiling point elevation of the solution under atmospheric pressure (°C)
Δ*P_b_*	transmembrane pressure differential (bar)
Δ*T*	temperature difference between the heat vapor inside tubes and the feed outside tubes (°C)
Δ*π*	osmotic pressure differentia (bar)
*ε*	compression ratio
*ε_p_*, *ε_VFD_*, *ε_bp_*	mechanical efficiency
*η_IP_*	efficiency of the water intake pump
*λ_d_*	latent heat of the steam (KW)
*λ_vp_*	latent heat below the demister (KW)
*μ*	dynamic viscosity
*ξ_re_*	replacement rate of membrane components
*ρ*	brine density(kg/m^3^)
*φ*	concentration polarization coefficient
*ω*_1_, *ω*_2_, *ω*_3_, *η*_1_, *η*_2_	coefficients in the regression equation
	
Subscript:	
*f*1	feed in the first stage of the RO unit
*f*2	feed in the second stage of the RO unit
*f*3	feed in the third stage of the RO unit
*HEX*	preheater
*r*1	brine in the first stage of the RO unit
*r*2	brine in the second stage of the RO unit
*r*3	brine in the third stage of the RO unit
